# Supplementary material for: Critical behavior of Fredenhagen-Marcu string order parameters at topological phase transitions with emergent higher-form symmetries
Source: npj Quantum Inf. 2025 May 9;11(1):74. doi: 10.1038/s41534-025-01030-z (PMC12064440; doi:10.1038/s41534-025-01030-z)
Supplement: Supplementary file 1 — Supplemenrary information [file 41534_2025_1030_MOESM1_ESM.pdf]

Supplemental Materials for “Critical behavior of Fredenhagen-Marcu string order parameters at topological phase transitions with emergent higher-form symmetries”

Wen-Tao Xu, Frank Pollmann and Michael Knap
[1]*Department of Physics, Technical University of Munich, 85748 Garching, Germany*
[2]*Munich Center for Quantum Science and Technology (MCQST), Schellingstr. 4, 80799 München, Germany*

CONTENTS

I. Energy density, correlation length, expectation values of local Hamiltonian terms and local order parameters 1

II. Discontinuity of the FM string order parameter in the absence of an emergent 1-form symmetry 2

III. Dual FM string order parameter and the stability 3

IV. Equivalence between the FM string order parameters defined using contractible and non-contractible loops 4

V. Analysis of the FM string order parameter for the deformed toric code state 6

I. ENERGY DENSITY, CORRELATION LENGTH, EXPECTATION VALUES OF LOCAL HAMILTONIAN TERMS AND LOCAL ORDER PARAMETERS

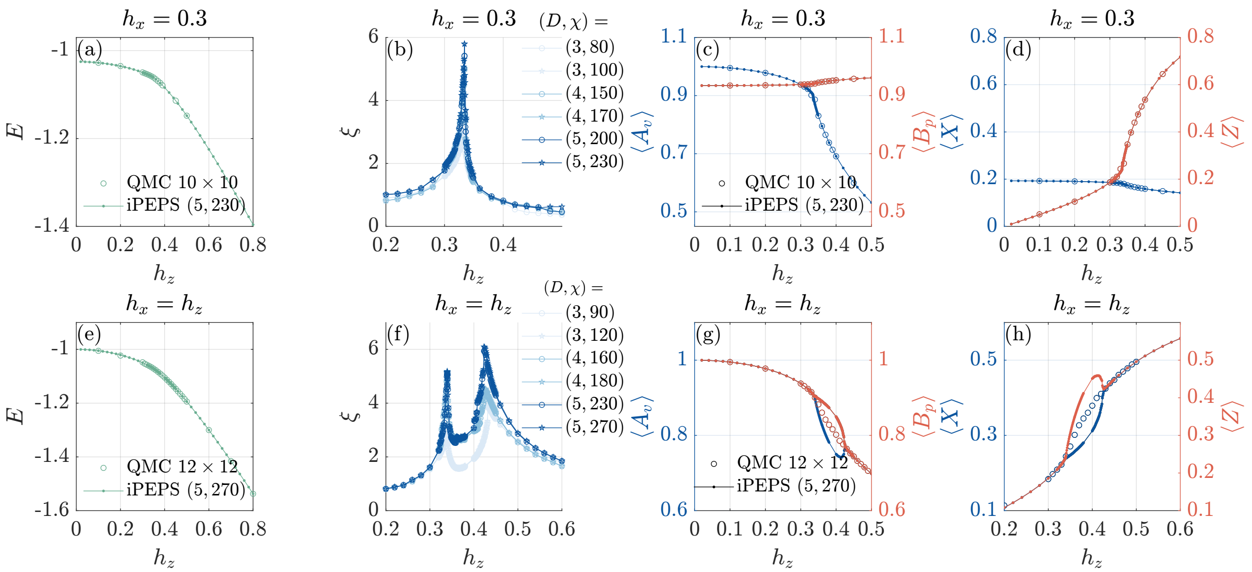

FIG. S1. **Comparison of ground state energy density and expectation values of local Hamiltonian terms from variational iPEPS and QMC.** (a) Ground state energy density along $h_x = 0.3$, the legend shows the bond dimensions (D, χ) of iPEPS and the system size of the QMC. (b) Correlation length from iPEPS with various bond dimensions along $h_x = 0.3$. (c) Expectation values of A_v and B_p along $h_x = 0.3$. (d) Expectation values of X and Z along $h_x = 0.3$. (e) Ground state energy density along the self-dual line $h_x = h_z$. (f) Correlation length from iPEPS with various bond dimensions along the self-dual line. (g) Expectation values of A_v and B_p along the self-dual line line. (h) Expectation values of X and Z along the self-dual line.

In this section, we benchmark the ground state energy density and the expectation values of local terms of the toric code model by comparing the ground state energy density and expectation values of local Hamiltonian terms from our optimized iPEPS and quantum Monte Carlo (QMC) simulations [1]. First, we consider the line $h_x = 0.3$. The results obtained from iPEPS match perfectly with those from the quantum Monte Carlo (QMC) [1], as shown in Figs. S1a, c and d. Extrapolating the peak positions of correlation length $h_{zc}^{(J)}(D)$ (see Fig. S1b) obtained from the iPEPS with different bond dimensions using a function $h_{zc}^{(J)}(D) = a/D^b - h_{zc}^{(J)}$, where we ignore the χ dependence and a, b are parameters, we can roughly determine that the phase transition point J shown in Fig. 1a is $h_{zc}^{(J)} = 0.335(1)$, which is close to result $h_{zc}^{(J)} = 0.333(1)$ of Ref. [1].

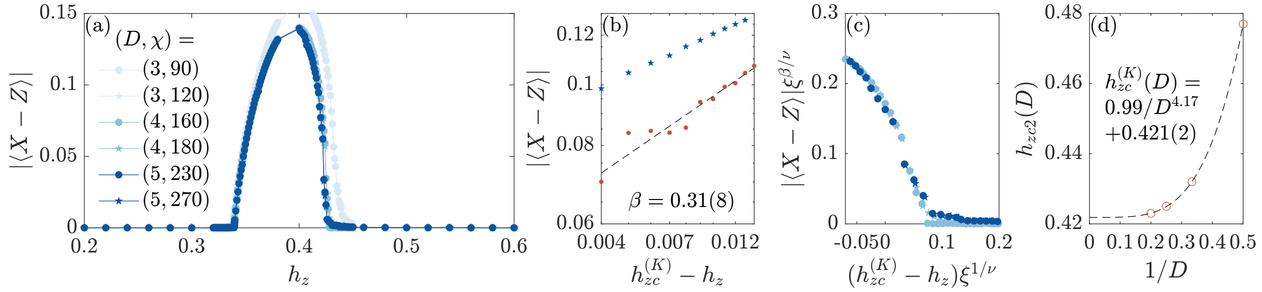

FIG. S2. **Local order parameter along the self-dual line ($h_x = h_z$) and its scaling near the critical endpoint K of variational iPEPS.** (a) Result from iPEPS with various bond dimensions. (b) Double-log plot extracting the critical exponent β_{local} of $|\langle X - Z\rangle|$ by a linearly extrapolation in $1/D$ (red dots), where $h_{zc}^{(F)} = 0.421(2)$. The blue stars are $|\langle X - Z\rangle|$ from iPEPS with the bond dimensions $(D, \chi) = (5, 270)$. (c) Data collapse of the local order parameter at the critical endpoint K, where $\nu = 0.629970(4)$ and $\beta = 0.326418(2)$. (d) Extrapolating the position of the critical end point K from the peak positions of the correlation length in Fig. S1f.

In Figs. S1e, g and h, we compare the energy density and expectation values along the self-dual line ($h_x = h_z$) with the QMC result. The correlation length shown in the inset of Fig. S1f has two peaks corresponding to the multi-critical point M and the critical endpoint K. Different from finite size QMC simulation where the symmetries can not be broken spontaneously, we can obtain $\langle A_v\rangle \neq \langle B_p\rangle$ and $\langle X\rangle \neq \langle Z\rangle$ for intermediate fields from the iPEPS results, implying a spontaneous duality symmetry breaking. Using the same method for extrapolating the position of J, we can roughly determine that the multi-critical point M shown in Fig. 1a is $h_{zc}^{(M)} = 0.3397(2)$, which is close to $h_{zc}^{(M)} = 0.340(2)$ of Ref.[1] and $h_{zc}^{(M)} = 0.3406(4)$ of Ref. [2]. Moreover, the position of the critical endpoint K strongly depends on the bond dimension D. As shown in the inset of Fig. S2d, the extrapolated position of K is $h_{zc}^{(K)} = 0.421(2)$, which is close to $h_{zc}^{(K)} = 0.418(2)$ of Ref. [1] and indicates that $h_{zc}^{(K)} = 0.48(2)$ obtained in Ref. [2] is questionable.

It is natural to expect that the phase transition at the critical endpoint K is also described by the 3D Ising universality class according to the universality hypothesis [2, 3], because it is a conventional spontaneous $\mathbb{Z}_2$ symmetry breaking phase transition in (2 + 1)D. It is interesting to check the universality hypothesis using the iPEPS simulation results in Fig. S2a. Using the same method for extracting other critical exponents, we obtain $\beta_{\text{local}} = 0.31(8)$ defined by $|\langle X - Z\rangle| \sim (h_{zc}^{(K)} - h_z)^{\beta_{\text{local}}}$, as shown in the inset of Fig. S2b; since extrapolated results have large fluctuation, we also show $|\langle X - Z\rangle|$ from iPEPS with bound dimensions (5, 270). So $\beta_{\text{local}} = 0.31(8)$ is close to $\beta = 0.326418(2)$ from the 3D Ising universality class [4]. Moreover, it can be found that the data from $D > 3$ can collapse; see Fig. S2c. These results imply that the critical endpoint K is consistent with the 3D Ising universality class.

II. DISCONTINUITY OF THE FM STRING ORDER PARAMETER IN THE ABSENCE OF AN EMERGENT 1-FORM SYMMETRY

We have shown that the FM string order parameter is discontinuous in the confined phase of the deformed toric code wave fucntion, which to some extend is fine tuned. However, it is unclear whether a similar behavior is expected for the variational iPEPS. Directly evaluating the FM string order parameter from the variational iPEPS in the confined phase is not possible due to numerical instability. Instead, we compute a perturbed wavefunction near the infinite large field limit, which does not suffer from numerical instabilities. To this end, we reparameterize the toric code Hamiltonian with $h_x = r\cos(\theta), h_z = r\sin(\theta)$ and apply a unitary transformation

$$U = \begin{pmatrix} \cos(\theta/2) & \sin(\theta/2) \\ -\sin(\theta/2) & \cos(\theta/2) \end{pmatrix} \tag{S1}$$

to it

$$U^\dagger H_{\text{TC}} U = -\sum_v A'_v - \sum_p B'_p - r\sum_e X_e, \tag{S2}$$

where $A'_v = \prod_{e\in v} X'_e$, $B'_p = \prod_{e\in p} Z'_e$, $X' = X\cos\theta - Z\sin\theta$ and $Z' = Z\cos\theta + X\sin\theta$. We first calculate in the U-transformed basis and then transform back to the original basis. When $r \to \infty$, the ground state of H_{TC} can be written as

$$\left|\Psi^{[0]}\right\rangle = \prod_e |\theta\rangle_e, \quad |\theta\rangle = U|+\rangle = \cos\frac{\theta}{2}|+\rangle + \sin\frac{\theta}{2}|-\rangle. \tag{S3}$$

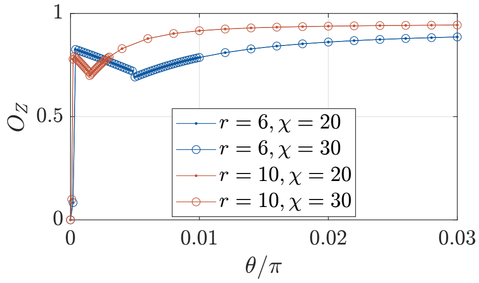

FIG. S3. **FM string order parameter in the confined region of a perturbatively constructed iPEPS.** The FM string order parameter evaluated using the iPEPS $|\tilde{\Psi}^{[1]}\rangle$ in Eq. (S7) constructed from first-order perturbation theory around the infinite field limit $r \to \infty$. χ is the bond dimension of the CTMRG environment.

The FM string order parameter evaluated in this limit is 1 for $\theta \neq 0$ and 0 for $\theta = 0$.

Near the infinite field limit, the first order ground state can be written as

$$\left|\Psi^{[1]}\right\rangle = \prod_e U_e \left(1 + \sum_v \prod_{e\in v} \sum_{\{\alpha_e=0,1\}} f_v(\{\alpha_e\}, r, \theta) Z_e^{\alpha_i} + \sum_p \prod_{e\in p} \sum_{\{\alpha_e=0,1\}} f_p(\{\alpha_e\}, r, \theta) Z_e^{\alpha_i}\right) \prod_e |+\rangle_e\,, \tag{S4}$$

where

$$f_v(\{\alpha_e\}, r, \theta) = \begin{cases} \left[\prod_{e=1}^4 (\cos\theta)^{1-\alpha_e}(-\sin\theta)^{\alpha_e}\right] / \left[2r\sum_{e=1}^4 \alpha_e\right], & \text{if } \sum_{e=1}^4 \alpha_e \neq 0; \\ 0, & \text{else if } \sum_{e=1}^4 \alpha_e = 0; \end{cases} \tag{S5}$$

$$f_p(\{\alpha_e\}, r, \theta) = \begin{cases} \left[\prod_{e=1}^4 (\sin\theta)^{1-\alpha_e}(\cos\theta)^{\alpha_e}\right] / \left[2r\sum_{e=1}^4 \alpha_e\right], & \text{if } \sum_{e=1}^4 \alpha_e \neq 0; \\ 0, & \text{else if } \sum_{e=1}^4 \alpha_e = 0. \end{cases} \tag{S6}$$

We can slightly change $|\Psi^{[1]}\rangle$ to $|\tilde{\Psi}^{[1]}\rangle$, such that it can be written as an iPEPS, and the difference between $|\tilde{\Psi}^{[1]}\rangle$ and $|\Psi^{[1]}\rangle$ is $O(1/r^2)$ [5]:

$$\left|\tilde{\Psi}^{[1]}\right\rangle = \prod_e U_e \left\{\prod_v \left[1 + \prod_{e\in v} \sum_{\{\alpha_e=0,1\}} f_v(\{\alpha_e\}, r, \theta) Z_e^{\alpha_i}\right] \prod_p \left[1 + \prod_{e\in p} \sum_{\{\alpha_e=0,1\}} f_p(\{\alpha_e\}, r, \theta) Z_e^{\alpha_i}\right]\right\} \prod_e |+\rangle_e\,. \tag{S7}$$

Now, $|\tilde{\Psi}^{[1]}\rangle$ can be interpreted as some gates applies on a product state, and it can be easily written as a 2×2 unit cell iPEPS with a bond dimension 2. As a next step, we evaluate the FM string order parameter from the iPEPS of $|\tilde{\Psi}^{[1]}\rangle$; see Fig. S3. It is singular near (but not exactly at) the h_x axis and displays non-analytical behavior for some finite θ. This implies that also for the variational iPEPS the FM string order parameter cannot be applied in the absence of an underlying associated 1-form symmetry.

III. DUAL FM STRING ORDER PARAMETER AND THE STABILITY

Because of the electric-magnetic duality of the toric code model, there is a dual FM string order parameter O_X, defined by replacing Z with X as well as the loop (string) on the primal lattice with those on the dual lattice in the FM string order parameter:

$$O_X = \lim_{|\hat{L}_{x,1/2}|\to\infty} \sqrt{|C_X(|\hat{L}_{x,1/2}|)|}, \qquad C_X(|\hat{L}_{x,1/2}|) = \frac{\langle\Psi|\prod_{e\in\hat{L}_{x,1/2}} X_e|\Psi\rangle / \langle\Psi|\Psi\rangle}{\sqrt{\langle\Psi|\prod_{e\in\hat{L}_x} X_e|\Psi\rangle / \langle\Psi|\Psi\rangle}}, \tag{S8}$$

where $\hat{L}_x$ is a non-contractible loop on the dual lattice whose length is twice the length of the string $\hat{L}_{x,1/2}$ on the dual lattice. Along the self-dual line, we expect that both O_X and O_Z are zero. However, the proper ground state needs to be chosen to obtain the desired results.

In Fig. S4a, we calculate both O_X and O_Z using a ground state $|0_x 0_y\rangle$ (the simultaneous ground state of the emergent Wilson operators on the non-contractible loops in x and y directions) that spontaneously breaks the emergent 't Hooft loop symmetry. It can be found that the O_Z is zero in the toric code phase as expected, but O_X is non-zero and unstable in the toric code phase, indicating that the FM string order parameters are unstable when the corresponding emergent 1-form symmetries are absent.

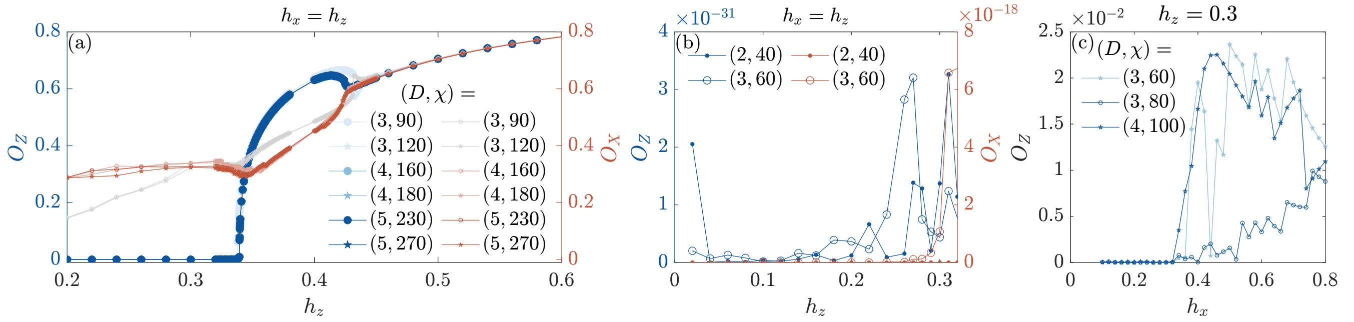

FIG. S4. **FM string order parameter and its dual of the variational iPEPS.** (a) Along the self-dual line, the FM string order parameter and its dual from iPEPS that respects the Wilson loop symmetry but spontaneously breaks the 't Hooft loop symmetry with various bond dimensions. The toric code phase, the duality symmetry breaking phase, and the trivial phase arise with increasing field at non-analytic points of the FM order parameter. (b) Along the self-dual line, the FM string order parameter and its dual evaluated using the trivial minimally entangled state in the toric code phase. Both of them are zero (to machine precision). (c) The FM string order parameter O_Z calculated along the line $h_z = 0.3$. Its dual O_X along $h_z = 0.3$ is equivalent to O_Z along $h_x = 0.3$, which is shown in Fig. 2a of the main text. Due to the absence of the Z 1-form symmetry in the confined regime, the FM string order parameter O_Z cannot be used to infer the properties of the critical point.

If we want to obtain the correct behavior of both O_X and O_Z from the same ground state, we can use the minimally entangled state. At $h_x = h_z = 0$, the iPEPS tensor A has a virtual $\mathbb{Z}_2$ symmetry, see Fig. 6a in the main text. The virtual $\mathbb{Z}_2$ symmetry allows us to construct the Wilson loop operators at the virtual level [6], which are equivalent to the Wilson loop operators on the physical level used to obtain all degenerate ground states. To obtain the minimally entangled states away from the limit $h_x = h_z = 0$, we have to impose the virtual $\mathbb{Z}_2$ symmetry to the iPEPS tensor, see Fig. 5b in the main text. We find that the ground state energies obtained from the iPEPS with and without imposing virtual $\mathbb{Z}_2$ symmetry are very close to each other in the toric code phase for various bond dimensions. So, we can safely impose the virtual $\mathbb{Z}_2$ symmetry to the iPEPS tensor in the toric code phase, which corresponds to the emergent Wilson loop symmetry or the emergent 't Hooft loop symmetry on the physical level. We can apply a projector $\lim_{N\to\infty}(\mathbb{1}_D^{\otimes N} + Z_D^{\otimes N})/2$ (see Z_D in Fig. 5b of the main text) to the virtual level of iPEPS to project to the minimally entangled state in the trivial topological sector, see detail in Appendix G of Ref. [7]. We find that both O_X and O_Z become zero when the ground state is chosen as the trivial minimally entangled state; see Fig. S4b. This agrees with the FM string order parameters whose denominators are defined using contractible loops.

In the trivial phase, there are two degenerate ground states in the duality symmetry-breaking phase corresponding to the predominant condensation of charges and fluxes, respectively. In Fig. S4a, we use the one with predominant condensation of charges, so the FM string order parameter O_Z detecting the charge condensation is well-behaved. However, the dual FM string order parameter O_X detects the flux condensation is discontinuous at the multi-critical point M because $O_X = 0$ is in the toric code phase if we use the trivial minimally entangled state. since the charge condensation predominant ground state does not have the emergent 1-form 't Hooft loop symmetry, it implies that the corresponding emergent 1-form symmetries are necessary for the FM string order parameters to be continuous at second-order phase transitions and exhibit proper criticality.

We further investigate the behavior of O_Z along a path $h_z = 0.3$ which crosses the transition between the deconfined phase and the confined regime, where we initialize the iPEPS using the state $|0_x 0_y\rangle$ and do not impose the virtual $\mathbb{Z}_2$ symmetry. As show in Fig. S4c, $O_Z = 0$ in the deconfined phase as we expected. However, it becomes unstable in the confined regime because the denominator of the FM string order parameter decays too fast to zero with the length of the loop, and this instability becomes more severe with h_x increasing. Because in the confined regime, in which an underlying Z 1-form symmetry does not exist, the FM string order parameter O_Z cannot capture the critical point between the deconfined phase and the confined regime.

IV. EQUIVALENCE BETWEEN THE FM STRING ORDER PARAMETERS DEFINED USING CONTRACTIBLE AND NON-CONTRACTIBLE LOOPS

In this section, we show that the FM string order parameters defined using a non-contractible loop L_x and evaluated using the trivial minimally entangled state and the one defined using a contractible loop L are equivalent, provided that the strings are infinitely long and the ground state is an iPEPS. First, consider the case with a contractible loop. According to Eq. (3) in the

main text, we need to consider the following three infinitely large tensor networks:

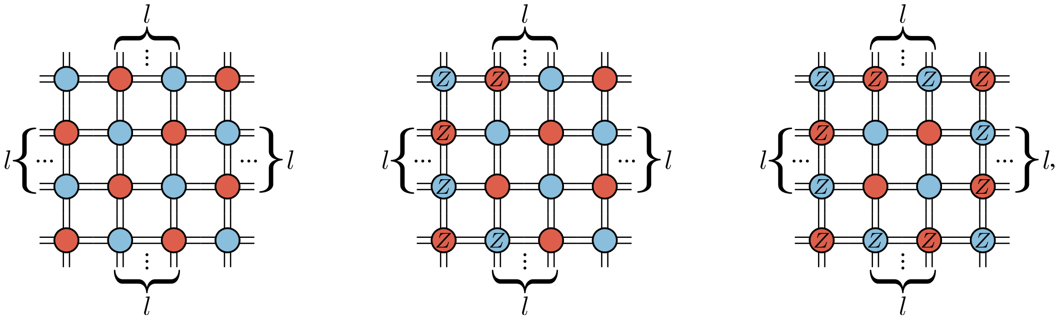

(S9)

where the length of the loop and the string are $|L| = 4l + 4$ and $|L_{1/2}| = 2l + 2$, respectively. When l is very large, using the edge tensors from the CTMRG, one can replace the middle part with the object shown on the right hand side of the following equation:

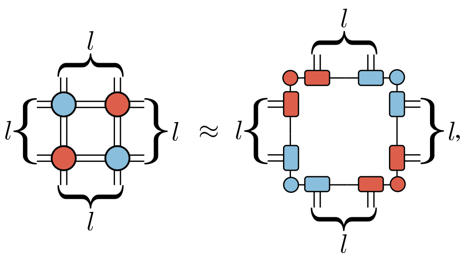

(S10)

where the corner tensors (not CTMRG corner tensor) represented by circles can be obtained using a method shown in Ref. [8]. However, we do not need to calculate the corner tensors represented by circles because they will be canceled later. Since the iPEPS tensors have the virtual $\mathbb{Z}_2$ symmetry shown in Fig. 5b, the right hand side of Eq. (S10) should also have this symmetry:

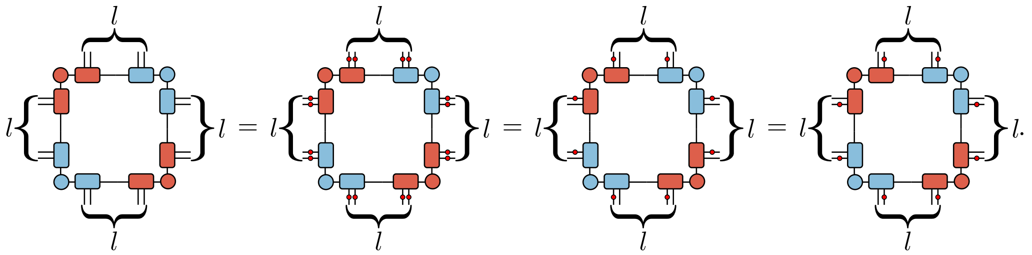

(S11)

Notice it is not always guaranteed that the object on the right side of Eq. (S10) has the virtual $\mathbb{Z}_2$ symmetry because the environment of the iPEPS could spontaneously break the virtual $\mathbb{Z}_2$ symmetry [9]. If so, we must apply a projector $(\mathbb{1}^{\otimes N} + Z_D^{\otimes N})/2$ to the object to restore the virtual $\mathbb{Z}_2$ symmetry, similar to what we do for obtaining the trivial minimally entangled state [10, 11]. With the object in the right side of Eq. (S10) as well as the corner and edge tensors of the CTMRG environment, the three infinity tensor networks in Eq. (S9) can be approximated as:

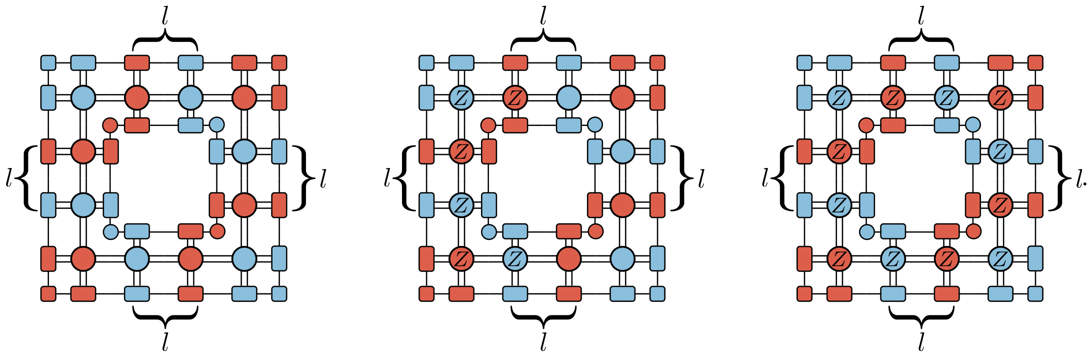

(S12)

Because l is very large, we can replace the power of transfer matrices $\mathcal{T}$ and $\mathcal{T}_Z$ with their fixed points to simplify the three tensor networks in Eq. (S12):

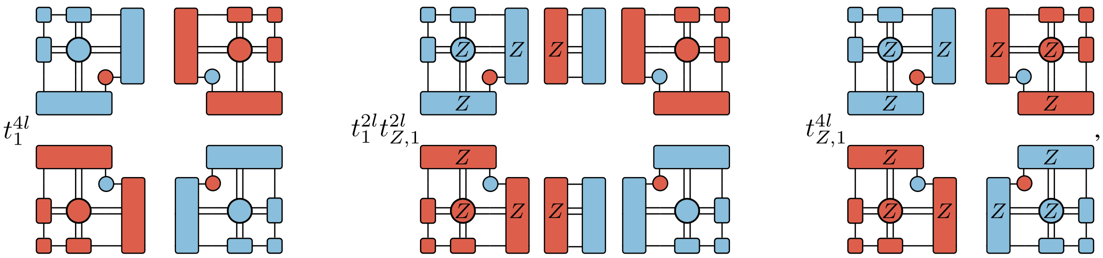

(S13)

where we assume that the dominant eigenvectors are not degenerate. We denote the corner objects as

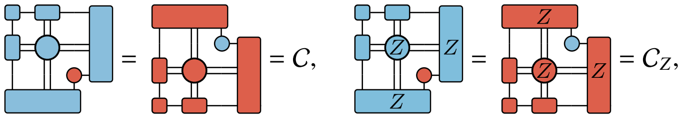

(S14)

where the symmetry of the square lattice is taken into consideration. With Eqs. (S13) and (S14), we can express the FM string order parameter as

$$O_Z = \left(\frac{t_1^{2l} t_{Z,1}^{2l} C^2 C_Z^2 |\langle V_Z | V \rangle|^2 / (C^4 t_1^{4l})}{\sqrt{(C_Z^4 t_{1,Z}^{4l})/(C^4 t_1^{4l})}} \right)^{1/2} = \left(\frac{t_1^{2l} t_{Z,1}^{2l} C^2 C_Z^2 |\langle V_Z | V \rangle|^2}{C_Z^2 C^2 t_{1,Z}^{2l} t_1^{2l}} \right)^{1/2} = |\langle V_Z | V \rangle|. \tag{S15}$$

Comparing with the calculation of the FM string order parameter defined using a non-contractible loop, we can conclude that the FM string order parameters whose denominators are defined using a non-contractible loop and evaluated using the trivial minimally entangled state and the one defined using a contractible loop are equivalent in the limit $|L_{1/2}| \to \infty$. When the symmetry depicted in Eq. (S11) is not satisfied, we should take all four diagrams in Eq. (S11) separately into account. In the presence of degenerate dominant eigenvectors Eq. (S13) is not valid. Nonetheless, using the virtual $\mathbb{Z}_2$ symmetry of the iPEPS tensor and the condition shown in Eq. (S23), we can still arrive at the same conclusion. We do not show this more complicated case here.

V. ANALYSIS OF THE FM STRING ORDER PARAMETER FOR THE DEFORMED TORIC CODE STATE

Here we discuss the relation between the FM string order parameter and the virtual order parameter defined for a topological iPEPS [12] and the origin of the discontinuity of the FM string order parameter without bulk phase transition. First, let us review the definition of the virtual order parameter. For a fixed point ground state, we can create a pair of charge excitations at the vertices v and v' by inserting two Z operators at the virtual level, because it is equivalent to Z operators along the string $L_{1/2}$ on the physical level:

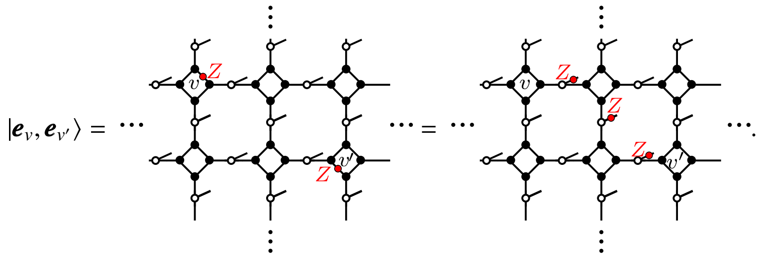

(S16)

Then, the virtual order parameter can be expressed as

$$O_Z^{(\mathrm{virtual})} = \lim_{|L_{1/2}| \to \infty} \left(\frac{\langle \mathrm{TC} | \prod_e Q_e^2(g_x, g_z) | \boldsymbol{e}_v, \boldsymbol{e}_{v'} \rangle}{\langle \mathrm{TC} | \prod_e Q_e^2(g_x, g_z) | \mathrm{TC} \rangle} \right)^{1/2}. \tag{S17}$$

In contrast, the FM string order parameter can be expressed as

$$O_Z = \lim_{|L_{L/2}|\to\infty} \left\{ \frac{\langle \mathrm{TC}| \left[\prod_e Q_e(g_x,g_z)\right] \left(\prod_{e\in L_{1/2}} Z_e\right) \left(\prod_e Q_e(g_x,g_z)\right] |\mathrm{TC}\rangle}{\sqrt{\langle \mathrm{TC}| \left[\prod_e Q_e(g_x,g_z)\right] \left(\prod_{e\in L} Z_e\right) \left[\prod_e Q_e(g_x,g_z)\right] |\mathrm{TC}\rangle}} \right\}^{1/2}$$

$$= \lim_{|L_{1/2}|\to\infty} \left\{ \frac{\langle \mathrm{TC}| \left[\prod_e Q_e(g_x,g_z)\right] \left[\prod_{e\in L_{1/2}} Q_e(g_x,g_z) Q_e(-g_x,g_z)\right] |\boldsymbol{e}_v, \boldsymbol{e}_{v'}\rangle}{\sqrt{\langle \mathrm{TC}| \left[\prod_{e\notin L} Q_e^2(g_x,g_z)\right] \left[\prod_{e\in L} Q_e(g_x,g_z) Q_e(-g_x,g_z)\right] |\mathrm{TC}\rangle}} \right\}^{1/2}. \tag{S18}$$

When $g_x = 0$, the FM string order parameter and the virtual order parameter are equal: $O_Z = O_Z^{(\text{virtual})}$. When $g_x \neq 0$, the FM string order parameter has extra defect lines along the string $L_{1/2}$ in the numerator and the loop L in the denominator compared to the virtual order parameter. If g_x is not too large, the string operator $\prod_{e\in L_{1/2}} Z_e$ will create two charges at its two ends when applied to the deformed toric code state. Thus, the FM string order parameter and the virtual order parameter are quantitatively the same and the FM string order parameter detects the condensation of charges. However, if g_x is too large, the string operator $\prod_{e\in L_{1/2}} Z_e$ will fail to create the charges when applied to the deformed toric code state. The FM string order parameter becomes essentially different from the virtual order parameter.

We can understand when the string operator $\prod_{e\in L_{1/2}} Z_e$ fails to create charges from transfer matrices:

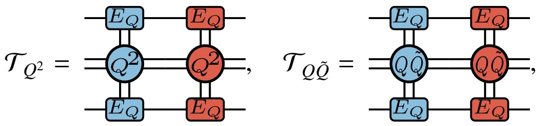

(S19)

where

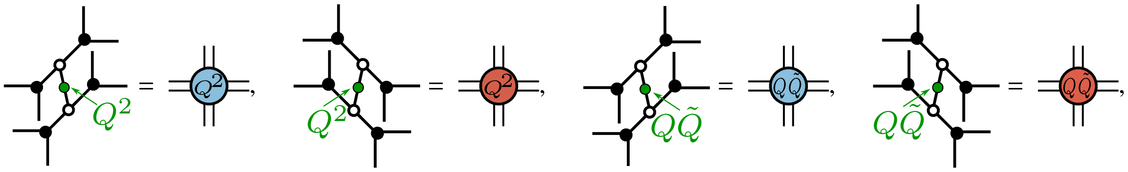
(S20)

$\tilde{Q}(g_x, g_z) = -Q(g_x, g_z)$, and E_Q is the boundary MPS tensor of the double tensor labeled by Q^2. Notice that the black and white dot tensors are defined in Fig. 6a. Using the transfer matrix fixed points, the virtual order parameter and FM string order parameter can be expressed as

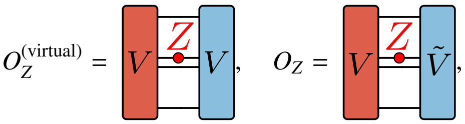

(S21)

where V and $\tilde{V}$ are fixed points of $\mathcal{T}_{Q^2}$ and $\mathcal{T}_{Q\tilde{Q}}$, respectively. Because $\mathcal{T}_{Q^2}$ and $\mathcal{T}_{Q\tilde{Q}}$ has a $\mathbb{Z}_2$ symmetry $U_X \otimes X \otimes X \otimes U_X$ in the toric code phase and the flux condensation phase (this symmetry does not exist in the charge condensation phase because the boundary MPS generated by E_Q spontaneously breaks the virtual $\mathbb{Z}_2$ symmetry [9]):

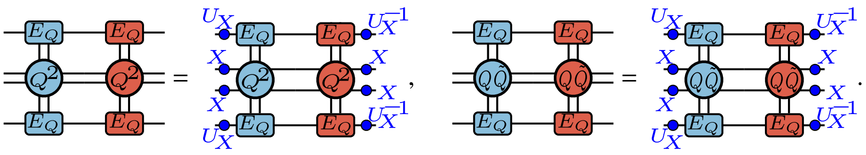
(S22)

where U_X is a $\chi \times \chi$ matrix defined via

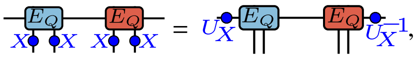
(S23)

we say the parity of V and $\tilde{V}$ is even (odd) if they are eigenstates of $U_X \otimes X \otimes X \otimes U_X$ with eigenvalues 1 (-1). It can be checked that V is always parity even in the toric code phase and the flux condensation phase, so $O_Z^{(\text{virtual})} = 0$ because of Eq. (S21) and $\{U_X \otimes X \otimes X \otimes U_X, \mathbb{1}_\chi \otimes Z \otimes \mathbb{1}_2 \otimes \mathbb{1}_\chi\} = 0$. However, along to $g_x^2 + g_z^2 = 0.65^2$ shown in Figs. 4a and f, $\tilde{V}$ is parity even (odd) when $\theta \gtrsim 0.4\pi$ ($0.25\pi < \theta \lesssim 0.4\pi$), so $O_Z = 0$ ($O_Z \neq 0$) when $\theta \gtrsim 0.4\pi$ ($0.25\pi < \theta \lesssim 0.4\pi$) according to Eq. (S21). Therefore, in

the flux condensation phase, the abrupt change of parity of $\tilde{V}$ is the origin of the discontinuity of the FM string order parameter in the flux condensation phase.

[1] F. Wu, Y. Deng, and N. Prokof'ev, Phase diagram of the toric code model in a parallel magnetic field, Phys. Rev. B **85**, 195104 (2012).

[2] J. Vidal, S. Dusuel, and K. P. Schmidt, Low-energy effective theory of the toric code model in a parallel magnetic field, Phys. Rev. B **79**, 033109 (2009).

[3] A. M. Somoza, P. Serna, and A. Nahum, Self-dual criticality in three-dimensional $\mathbb{Z}_2$ gauge theory with matter, Phys. Rev. X **11**, 041008 (2021).

[4] F. Kos, D. Poland, D. Simmons-Duffin, and A. Vichi, Precision islands in the ising and o (n) models, Journal of High Energy Physics **2016**, 1 (2016).

[5] L. Vanderstraeten, M. Mariën, J. Haegeman, N. Schuch, J. Vidal, and F. Verstraete, Bridging perturbative expansions with tensor networks, Phys. Rev. Lett. **119**, 070401 (2017).

[6] N. Schuch, I. Cirac, and D. Pérez-García, Peps as ground states: Degeneracy and topology, Annals of Physics **325**, 2153 (2010).

[7] L. Haller, W.-T. Xu, Y.-J. Liu, and F. Pollmann, Quantum phase transition between symmetry enriched topological phases in tensor-network states (2023), arXiv:2305.02432 [cond-mat.str-el].

[8] L. Vanderstraeten, J. Haegeman, P. Corboz, and F. Verstraete, Gradient methods for variational optimization of projected entangled-pair states, Phys. Rev. B **94**, 155123 (2016).

[9] J. Haegeman, V. Zauner, N. Schuch, and F. Verstraete, Shadows of anyons and the entanglement structure of topological phases, Nature communications **6**, 8284 (2015).

[10] L. Haller, W.-T. Xu, Y.-J. Liu, and F. Pollmann, Quantum phase transition between symmetry enriched topological phases in tensor-network states, Phys. Rev. Res. **5**, 043078 (2023).

[11] W.-T. Xu, M. Knap, and F. Pollmann, Entanglement of gauge theories: from the toric code to the $\mathbb{Z}_2$ lattice gauge higgs model (2023), arXiv:2311.16235 [cond-mat.str-el].

[12] G.-Y. Zhu and G.-M. Zhang, Gapless coulomb state emerging from a self-dual topological tensor-network state, Phys. Rev. Lett. **122**, 176401 (2019).
